# A Network-Based Systematic Study for the Mechanism of the Treatment of Zhengs Related to Cough Variant Asthma

**DOI:** 10.1155/2013/595924

**Published:** 2013-11-28

**Authors:** Di Chen, Fangbo Zhang, Shihuan Tang, Yan Chen, Peng Lu, Shaoxin Wen, Hongchun Zhang, Xi Liu, Enxiang Chao, Hongjun Yang

**Affiliations:** ^1^Institute of Automation, Chinese Academy of Sciences, Beijing 100190, China; ^2^Institute of Chinese Materia Medica, China Academy of Chinese Medical Sciences, Beijing 100700, China; ^3^China-Japan Friendship Hospital, Beijing 100029, China

## Abstract

Traditional Chinese medicine (TCM) has shown significant efficacy in the treatment of cough variant asthma (CVA), a special type of asthma. However, there is shortage of explanations for relevant mechanism of treatment. As Zhengs differentiation is a critical concept in TCM, it is necessary to explain the mechanism of treatment of Zhengs. Based on TCM clinical cases, this study illustrated the mechanism of the treatment of three remarkably relevant Zhengs for CVA: “FengXieFanFei,” “FeiQiShiXuan”, and “QiDaoLuanJi.” To achieve this goal, five steps were carried out: (1) determining feature Zhengs and corresponding key herbs of CVA by analyses of clinical cases; (2) finding out potential targets of the key herbs and clustering them based on their functional annotations; (3) constructing an ingredient-herb network and an ingredient network; (4) identifying modules of the ingredient network; (5) illustrating the mechanism of the treatment by further mining the latent biological implications within each module. The systematic study reveals that the treatment of “FengXieFanFei,” “FeiQiShiXuan,” and “QiDaoLuanJi” has effects on the regulation of multiple bioprocesses by herbs containing different ingredients with functions of steroid metabolism regulation, airway inflammation, and ion conduction and transportation. This network-based systematic study will be a good way to boost the scientific understanding of mechanism of the treatment of Zhengs.

## 1. Introduction

Traditional Chinese medicine (TCM), as a system of ancient medical practice which differs in substance, methodology, and philosophy from modern medicine, involves a broad range of empirical testing and refinement and plays an important role in the health maintenance for people. During the long history of TCM clinical practices, much treatment experience for a myriad of diseases has been accumulated [1]. The clinical cases of TCM, especially those from experienced TCM doctors, are precious sources for the understanding and development of TCM.

 Doctors diagnose whether a patient is suffering from certain types of diseases or not based on a comprehensive analysis of the patient's symptoms. In the field of TCM, it is especially important to identify patients' Zhengs based on the four diagnostic methods, which include inspection, listening and smelling, inquiry, and pulse taking. According to Zhengs, TCM practitioners will prescribe proper formulas to patients in order to heal their disorders [2, 3]. Herbal medicines, a powerful tool of TCM, can heal different kinds of Zhengs by combinations [4–6]. Although there are substantial records of TCM clinical cases and corresponding analyses of the clinical information [7–9], there is still shortage of scientific explanations for corresponding mechanism. 

With the development of bioinformatics and system biology [10–12], it is possible to analyze the molecular mechanism of drugs. Some system biology tools, with extremely high efficiency and molecular level representation, have been invented (e.g., MetaDrug (http://www.genego.com/)). They are widely used for the understanding of the complex functions of compounds [13–15]. With the help of system biology tools, herbal functions and the mechanism of treatment of Zhengs can be interpreted in some degree. 

Cough variant asthma (CVA), a type of asthma with main symptom of a dry, nonproductive cough [16–18], is affecting many people's health. It is sometimes called chronic cough to describe a cough that has lasted longer than six to eight weeks. Together with lung disease and other symptoms such as tachycardia and dyspnea [19–22], CVA is one major health burden in the area of respiratory medicine. In the field of TCM, CVA is usually related with the Zheng of “FengXie (wind-evil)” and is named as “FengKe” in Chinese. 

Although TCM has shown remarkable curative effects for CVA, the latent mechanism is still unknown. Current studies on TCM have provided few insights into the biological mechanism. The obscurity of the mechanism will hinder the development and clinical popularizing of TCM theory. A systematic study on the mechanism of the treatment of TCM Zhengs from the aspects of herb ingredients, ingredient targets, and their functions, as well as the complex relationships among them is extremely necessary. Besides, explanations for the mechanism of the treatment of CVA-related Zhengs can help us validate the reasonability of TCM theory and develop a deep understanding of the mechanism which is beneficial to new herbal medicine design. This study tried to illustrate the biological mechanism of the treatment of Zhengs related with CVA by analyzing the clinical cases.

## 2. Materials and Methods 

**Figure 1:**
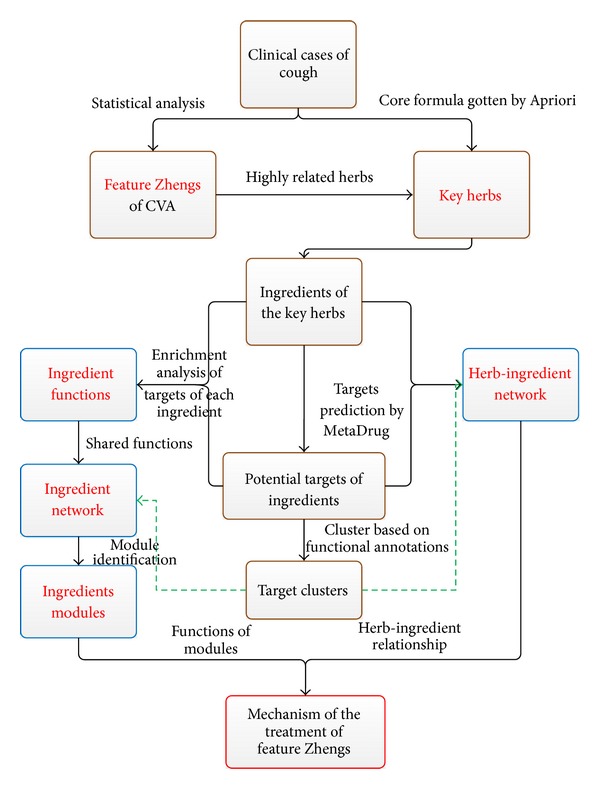
The flow diagram of the systematic study.

We have identified the potential mechanism of the herbs for the treatment of three distinguished Zhengs of CVA by a network-based systematic study (as shown in Figure 1) of the TCM clinical cases. Our approach proceeds as follows.

1. Determine feature Zhengs and corresponding key herbs of CVA by analyzing clinical cases.
2. Find out potential targets of key herbs and cluster them based on their functional annotations.
3. Construct an ingredient-herb network and an ingredient network to study the relationship between ingredients and herbs.
4. Identify modules in the ingredient network.
5. Illustrate the mechanism of the treatment by mining the latent biological implications (pathways and GOs [23]) within each module.

### 2.1. Analysis of TCM Clinical Cases

#### 2.1.1. Identification of Feature Zhengs of CVA

The information of the clinical cases was collected from June 2009 to July 2012, by the *Famous TCM Doctor Inheritance Studio of Chao Enxiang*, who has engaged in medical work for more than forty years and has accumulated many successful experiences of treating CVA [24, 25], and the data were exported from the *Traditional Chinese Medicine Inheritance Support Plat *[26]. These cases were recorded in standard format, so it is convenient to do analysis about them. These clinical cases contain the information of Zhengs, disease name, the principle and method of therapy, herbal formulae, patient's age, and gender, and so on. By statistical analyses of the clinical cases, we have identified some highly related Zhengs for CVA. These Zhengs are distinguished from others for they occur remarkably more times than the others in the clinical cases. These Zhengs, which represent the main feature of CVA, were defined as feature Zhengs of CVA.

#### 2.1.2. Identification of the Key Herbs

The clinical cases list the herbs which constitute the formulas. It is easy to find that similar Zhengs are treated by similar formulas as discussed in [27, 28]. Although formulas of patients with the same Zhengs can vary with individuals' conditions, there are always several herbs prescribed changelessly for the same Zhengs. The clinical cases from Professor Chao included 2414 cases and 948 records of formulas. These cases provided abundant information about the herb combination laws and treatment rules. As it is difficult to elaborate the mechanism of the treatment of Zhengs directly, we tried to illustrate the latent mechanism by finding the functions of the herbs alternatively. The conditional probability of an herb appeared in the cases given certain Zheng can be used to represent the correlation degree between an herb and a Zheng. In our study, more than one Zheng was identified as feature Zhengs; the conditional probability given these Zhengs is as below:

(1) $$\begin{array}{c} p\left(h\;|\;Z\right)=\frac{\sum_{i}c\left(h,z_{i}\right)}{\sum_{i}c\left(z_{i}\right)}, \end{array}$$

where *h* represents an herb, *Z* represents the set of feature Zhengs, *c*(*h*, *z*_*i*_) represents how many times herb *h* and Zheng *z*_*i*_ appear in the same case, and *c*(*z*_*i*_) is the times Zheng *z*_*i*_ appears in the clinical cases. *p*(*h* | *Z*) represents the probability of an herb that appeared given the feature Zhengs.

Herbs with high conditional probability are more related to the feature Zhengs. However, as TCM pays attention to the rules of composition, it is not suitable to consider each herb separately without consideration of their combination rules [29–31]. Apriori's algorithm [32] was used here to find out core formulas, and the minimum support was set at 0.4. Then herbs with high conditional probability and appearing in the core formulas at the same time were defined as the key herbs which were highly related to the feature Zhengs. By analyzing the mechanism of the key herbs, it will help to understand the mechanism of the treatment of feature Zhengs.

### 2.2. Potential Targets of Key Herbs

#### 2.2.1. Finding Out the Potential Targets

A drug target is a key molecule involved in particular metabolic or signaling pathways which are specific to a disease condition or pathology [33–35]. In order to find out the bioprocesses that may be affected by the herbs, we need to find out the potential targets of key herbs. If some targets are targeted by most of the key herbs, it is probable that these targets play key roles in the treatment of the feature Zhengs.

 To find out the potential targets of key herbs, we need to seek out the compounds of each herb at first. The targets of the ingredients of an herb were considered as the targets of this herb. In our study, we used MetaDrug, which is a system biology tool developed by the Gene GO company [14, 15], to find the potential targets of each compound. This tool uses the known targets of similar compounds to predict the possible targets of a compound. The similarity is referred to as the chemical structure similarity defined by Tanimoto's coefficient [36]. In order to achieve relatively reliable results, we set the lower and upper values of the coefficient to 0.9 and 1.0 to do the similarity search.

#### 2.2.2. Clustering the Potential Targets Based on Functions

Because of the great number of compounds of an herb and the multiple targets of each compound, the total number of the targets of all the key herbs is substantial. The great amount of targets makes it difficult to understand the biological meaning, so we clustered the targets according to their functional annotations. The clustering was done by DAVID [37], a tool widely used in the studies about genes or proteins. Targets in the same cluster share similar functional annotations, and different clusters represent different functions. 

### 2.3. Network-Based Analysis

#### 2.3.1. Herb-Ingredient Network

An herb-ingredient network is a bipartite network constructed by simply connecting an herb and its ingredients together. It can shed light on the relationship of different herbs' ingredients and supply some latent lines of evidence for the combination rules of herbs at the same time.

#### 2.3.2. Ingredient Network

We constructed an ingredient network based on the shared functions of ingredients.

1. Ingredient functions.

Since an ingredient achieves its effects by acting on certain targets, functions of an ingredient can be derived by the functions of its targets. However, one ingredient can have effects on multiple targets of which the functions are complex. We obtained the detailed functions of an ingredient by enrichment analyses of its targets in respect to GO processes, GO molecular functions, GO localizations, and pathways. The enrichment results were gotten from MetaDrug with a *P* value less than 1 × 10^−4^, and all of the four aspect functions are in standardized items. At last, each ingredient was assigned a standardized functional item set.

(2) Ingredient network construction.

According to the ingredient functions gotten from enrichment analyses of targets, the relationship between two ingredients can be represented by the intersection of their functional item sets. The weight of an edge on the ingredient network is defined as below:

(2) $$\begin{array}{c} w\left(c_{i},c_{j}\right)=\frac{1}{4}\cdot \sum_{k\in E}\frac{\left|F_{c_{i}}^{k}\cap F_{c_{j}}^{k}\right|}{\left|F_{c_{i}}^{k}\cup F_{c_{j}}^{k}\right|}, \end{array}$$

where *c*_*i*_, *c*_*j*_ are two ingredients; *E* represents four different enriched aspects: *E* = {*GO processes, GO molecular functions, GO localizations, pathways*}; *F*_*c*_*i*__^*k*^ represents the set of enriched functions of *c*_*i*_ in the aspect of *k*; |·| represents the size of a set; and ∩, ∪ represent intersection and union of two sets as usual.

We constructed an ingredient network in our study by only considering edges with a weight larger than the threshold of 0.2 and with *P* value (Fisher's exact test) less than 0.01. Besides, we also assigned the intersection of two sets of functional items: *F*_*c*_*i*__∩*F*_*c*_*j*__ to each edge (*c*_*i*_, *c*_*j*_) as its functional implications for further analysis, where *F*_*c*_*i*__ represents the united set of functional items of four aspects.

#### 2.3.3. Network Visualization

All of the networks were visualized by Cytoscape [38], a widely used network visualization tool. Targets, ingredients, and herbs in the networks are represented by nodes, and interactions are represented by edges.

### 2.4. Modules of Ingredient Network

To figure out the latent mechanism, just finding out functional relationship between ingredients by a network is not enough. We should also find out whether these ingredients collaborate with each other to achieve certain main functions or not. Modules [38, 39] are densely related parts of a network, so modules in the ingredient networks can stand for the functional related ingredients. We used MCODE [40], an effective network module identification plugin of Cytoscape, to identify the modules from the ingredient network. 

### 2.5. Module Functions

 Modules of the ingredient network can suggest which ingredients have similar functions. To explore the biological meaning hidden in each module, we employed the functional implications assigned to the edges, as discussed in Section 2.3.2. By counting the functional items of edges in each module, we got the frequent items whose frequencies were larger than half of the biggest counts of all items within a module to reveal the main functions of the module.

## 3. Results and Discussion

For thousands of years, TCM holds a great promise for medical treatments in China and now is considered as a complementary medical system in many Western countries [41]. Despite the fact that many positive outcomes have been observed in TCM clinical cases, the underlying mechanism of the treatment of Zhengs is unclear. An herbal formula usually incorporates several herbs which are chosen based on the patients' Zhengs, so we can catch sight of the underlying Zheng treatment mechanism by analyzing the related herbs indirectly. In this study, we applied our method to the clinical cases of Professor Chao to find out the biological mechanism.

### 3.1. Feature Zhengs Derived from Clinical Cases

**Figure 2:**
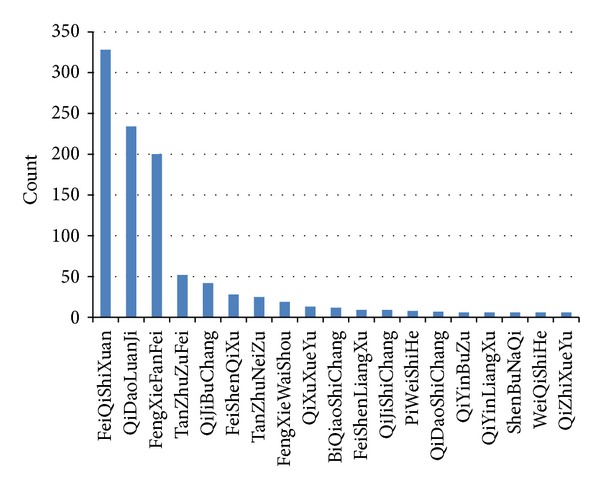
Count of Zhengs in the clinical cases. Among the clinical cases, there were 133 Zhengs in total. This histogram only shows the Zhengs with a count larger than 3. The horizontal axis represents different Zhengs, and the vertical axis represents the counts of one Zheng in the clinical cases.

We collected 2414 clinical cases of CVA-related diseases, and the final statistics showed that among all of the 133 different Zhengs recorded, “FengXieFanFei (wind-evil invading lung),” “FeiQiShiXuan (lung Qi obstruction),” and “QiDaoLuanJi (twin acute airway)” were three most frequent CVA-related Zhengs. As shown in Figure 2, these Zhengs were not only frequent, but also possessed an apparent advantage over all other Zhengs. These three Zhengs occupied 67% of all the Zhengs recorded in the clinical cases while the other 130 Zhengs occupied 33% of the cases. This manifested that most of the CVA cases were related to these three Zhengs. Consequently, “FeiQiShiXuan,” “QiDaoLuanJi,” “FengXieFanFei,” three most typical Zhengs for CVA, were determined as the feature Zhengs of these CVA-related clinical cases.

### 3.2. Key Herbs of the Feature Zhengs

#### 3.2.1. Conditional Probability of Each Herb Given the Feature Zhengs

**Figure 3:**
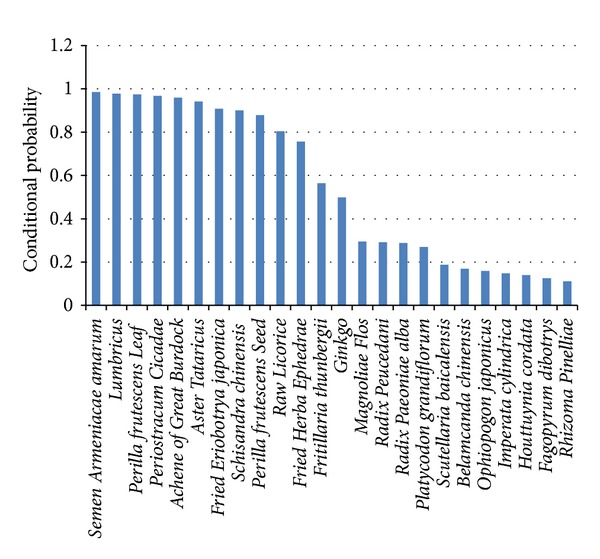
Conditional probability of an herb given feature Zhengs. This figure shows the conditional probability of an herb given feature Zhengs (“FeiQiShiXuan,” “FengXieFanFei,” and “QiDaoLuanJi”). Herbs with probability less than 0.1 are ignored.

To seek out the key herbs which are highly associated with the feature Zhengs, we calculated the probability as shown in (1) and got the results as shown in Figure 3.

As shown in Figure 3, the probabilities of the first 11 herbs—*Semen Armeniacae amarum* (SAA), *Lumbricus* (Lum), *Perilla frutescens Leaf* (PFL), *Periostracum cicadae* (PC), *Achene of Great Burdock* (AGB), *Aster tataricus* (AT), *Fried Eriobotrya japonica* (FEJ), *Schisandra chinensis* (SC), *Perilla frutescens seed* (PFS), *Raw Licorice* (RL), and *Fried Herba ephedrae* (FHE) were larger than 0.7. The probabilities of these 11 herbs are apparently higher than other herbs for the treatment of “FeiQiShiXuan,” “FengXieFanFei,” and “QiDaoLuanJi”; they must play key roles in the treatment of these feature Zhengs. 

#### 3.2.2. Core Formula

Furthermore, we applied Apriori's algorithm to determine the frequent herb combinations as the core formula for the treatment of feature Zhengs. The combinations found out by the algorithm (the support rate was set at 0.4) were SAA, Lum, PFL, PC, AGB, AT, FEJ, SC, PFS, RL, and FHE. 

The result showed that these 11 herbs were frequently prescribed together to heal CVA. Considering the high conditional probability of the 11 herbs as discussed in Section 3.2.1 and their frequent combination, these two results testified that not only do the 11 herbs play key roles by isolation analysis, but also they can combine with each other to achieve therapeutic effects. It is reasonable to define these 11 herbs as the key herbs of the feature Zhengs. There are also many studies that prove the effectiveness of the 11 herbs for the treatment of CVA [42–48], and nine of them are the ingredients of “Suhuangzhike” granules, a Chinese patent medicine which has shown significant clinical effects [49–51].

### 3.3. Potential Targets of Key Herbs

#### 3.3.1. Targets of the Herbs

There were 306 compounds collected for the 11 key herbs. The potential targets for the compounds were obtained by the targets prediction function of MetaDrug. The potential targets of an herb were considered as the sum of the targets of its compounds. The number of compounds and potential targets for each herb is listed in Table 1. This testifies the multicompounds and multitargets characteristics of Chinese herbs. There are 954 different targets for all 11 key herbs, and 372 of them are the targets of more than 7 compounds. To make our study more reliable and to show that Chinese medicine achieve the therapeutic effects by combination of compounds, we used the 372 targets which were targeted by more than 7 compounds for the following analyses.

#### 3.3.2. Target-Compound Network

**Figure 4:**
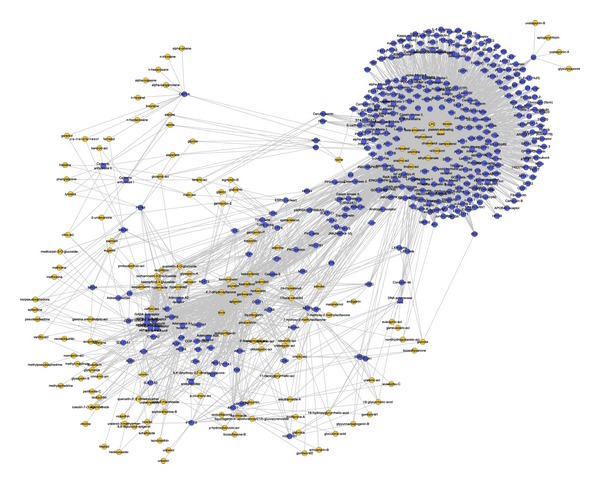
The interactions between potential targets and compounds. Yellow nodes represent the compounds, and the blue ones are potential targets.

We got the interaction networks of compounds and their potential targets as shown in Figure 4. As we can see from the network, multiple compounds act on the same targets. This is different from Western medicine which usually employs only one compound to act on one certain target.

#### 3.3.3. Clustering Targets Based on Functional Annotations

However, the number of targets is so big and the promiscuity of the targets restrains the understanding of the mechanism. To make clear the main functions of the key herbs, we clustered these targets by the functional annotations clustering function of DAVID. We got 18 clusters in total, and the main clusters are shown in Table 2. Clusters 1 to 3 are three biggest clusters; Clusters 4 and 5 are the clusters with OMIM [52] genes of asthma and cough, two similar diseases of CVA.

To find out the mechanism of the herbs, functions of these targets are of crucial roles. As the clustering process is based on functional annotations of each gene, different clusters must correspond to different functions. The functions of the biggest clusters correspond to the main functions of the key herbs. The genes in Cluster 1 are connected with hormone stimulus. Activated estrogen receptors (ER, Cluster 1) via ER*α* or ER*β* stimulate NO production in airway epithelium; estrogen-induced NO and its impairment may contribute to altered bronchodilator in women with asthma [53]. Peroxisome proliferator-activated receptor *γ* (PPAR*γ*, Cluster 1) ligands, such as rosiglitazone and pioglitazone, inhibit proliferation and inflammatory cytokine production from airway smooth muscles through a PPAR*γ*-independent mechanism [54]. The genes in Cluster 2 are associated with cellular metabolic process. Mitogen-activated protein kinase-1 (MAPK1, Cluster 2) controls a vast array of physiological processes. Inflammatory cytokines and environmental stresses may activate p38 MAPKs, which contributes to diseases like asthma and autoimmunity [55]. Pim kinases are a family of serine/threonine kinases that are induced by cytokines and involved in allergy and asthma. The inhibition of Pim1 kinase (Cluster 2) effectively prevents the development of airway hyperresponsiveness, airway inflammation, and cytokine production in allergen-sensitized and allergen-challenged mice [56]. Most studies of chronic cough or bronchial asthma deal with IgE responsiveness. Different serum IgG1, IgG2, and IgG3 (Immunoglobulin, Cluster 3) levels together with different numbers of peripheral blood eosinophil and CD8 lymphocytes represent different pathways of immune regulation of bronchial asthma [57].

The three biggest clusters are all related to the treatment of asthma or cough by regulating the hormone stimulus, airway inflammation, and immune regulations. Except for these three clusters, two clusters (Clusters 4, 5) have OMIM genes (Genes IDs: 718, 1080) of asthma or cough in the cluster, and we define the two clusters as disease-related target clusters. The genes in Cluster 4 are mainly related to complement system. The activation of the complement system could have a role in treating cough by promoting inflammation and enhancing airway hyperresponsiveness. C5a is usually elevated at the site of inflammation in patients with asthma [58]. The genes in Cluster 5 are related to transport function across cell membrane. ATP-binding cassette (ABC) transporters are a family of transmembrane proteins which can transport numerous substrates across biological membranes in an energy-dependent pattern. Many ABC transporters such as P-glycoprotein (P-gp), multidrug resistance-associated protein 1 (MRP1), and breast cancer resistance protein (BCRP) are highly expressed in bronchial epithelium. These three ABC transporters are well known to play an important role in lung functioning. Mutations in the cystic fibrosis transmembrane conductance regulator (CFTR) gene can cause cystic fibrosis. The role of altered function of ABC transporters in asthma has hardly been investigated so far [59].

The multiple functions of the targets prove that TCM aims not only to antagonize specific pathogenic targets, but also to correct maladjustments and restore the self-regulatory ability of the body. 

### 3.4. Herb-Ingredient Network

**Figure 5:**
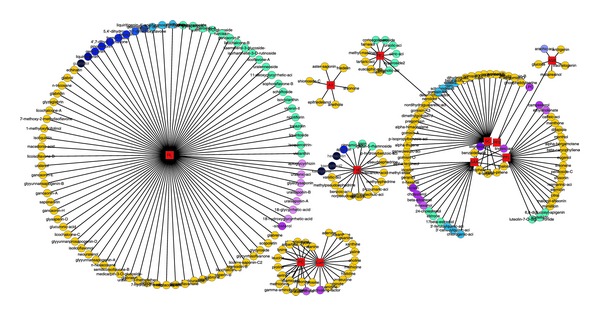
Herb-ingredient network. In this network, circular nodes are ingredients, square nodes are herbs, and an edge represents that an ingredient is a composition of an herb. Different colors stand for different target clusters. The shades of the colors represent the relation intensity of an ingredient to a disease-related target cluster. To be more specific, if the number of targets in Cluster 4 is larger than targets in Cluster 5, the ingredient is assigned purple, and the ingredient is assigned blue if the number of targets in Cluster 5 is larger. The shade of the color is proportional to the absolute value (ranging from 1 to 7) of the difference of the number of targets in Cluster 4 and Cluster 5. The only ingredient, with an absolute value of zero, was colored as bluish violet. Ingredients with targets which are not in disease-related target clusters are yellow, and those ones without any targets are not shown in this network. As the ingredients were hardly collected, they are not the overall compositions, but they can elucidate the problem in some degree.

To understand the mechanism of herb combinations, it is necessary to know whether ingredients of an herb have similar functions or different herbs share similar compositions. The herb-ingredient network, as shown in Figure 5, in which different colors represent different functions, provides a way to make clear the composition characteristics of herbs. 

 It is obvious to see from the herb-ingredient network that different key herbs used for CVA basically do not have same ingredients with only a few expectations, and nearly every herb has targets (targets with blue or purple color) of the disease-related target clusters. By combination of different herbs, more ingredients can collaborate with each other to achieve the same therapeutic effects, for ingredients of different herbs share similar functions.

### 3.5. Ingredient Network

#### 3.5.1. Ingredient Network Construction

**Figure 6:**
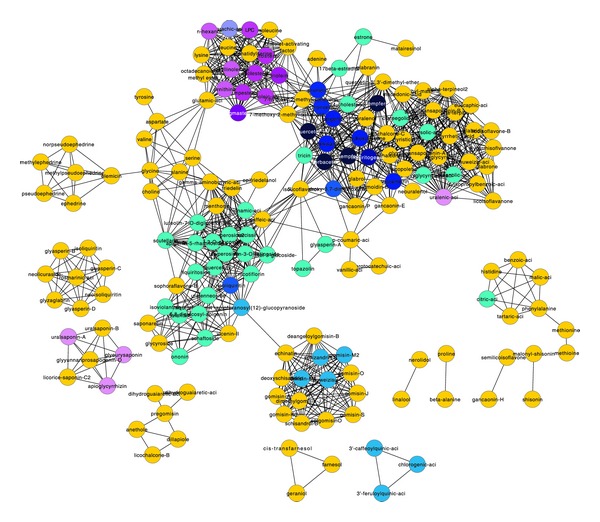
Ingredient network. The meanings of colors are the same as in Figure 5, the same in Table 3.

We constructed an ingredient network (Figure 6) to do further analysis of the relationship between herbs and ingredients. The edges of this network were decided by the intersection of the functional items enriched by the targets of two ingredients instead of the intersections of simple targets of two ingredients, because different targets, which can share similar functions by involving in the same processes or different pathways, do not stand for different functions of ingredients.

By construction of ingredient network, not only did we know whether there were interactions between ingredients, but also we obtained the implications of their interactions as partly shown in Table 3. For example, there is an interaction between the ingredients schaftoside and glycyroside, and the targets of two compounds share part of the enriched GOs and pathways, like the GO processes of “cellular aldehyde metabolic process,” GO molecular functions of “alcohol dehydrogenase (NADP+) activity,” and the pathway of “triacylglycerol metabolism p.1.” That is to say, ingredients schaftoside and glycyroside act on the targets with similar functions, in the same biological processes and in the same localizations of human body. Above all, each edge has corresponding functional annotations, which can reveal the interactions of two ingredients, with respect to GO processes, GO molecular functions, and GO localizations and pathways.

#### 3.5.2. Modules of Ingredient Network

**Figure 7:**
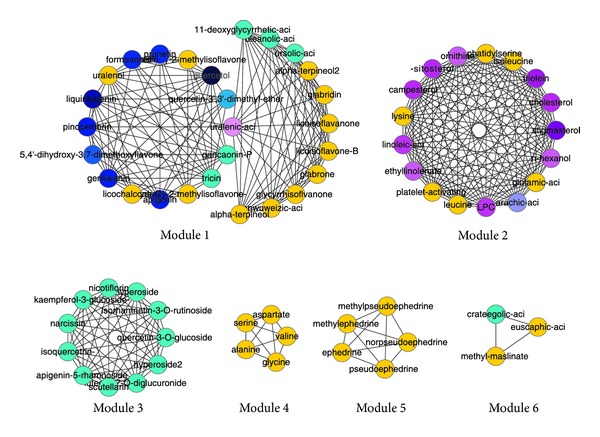
Modules of the ingredients network. The blue and purple nodes are in different modules in general, in accordance with the expectation that ingredients of different functions are mainly located in different modules. Then large modules should be in accordance with the main functions of key herbs. Module 1 is mainly composed of ingredients whose targets are not in the disease-related target clusters. Module 2 and Module 3 are composed of ingredients whose targets are mainly in the disease-related target Cluster 4 and Cluster 5, respectively.

Six modules (as shown in Figure 7) were identified from the ingredient network by the MCODE method; each module represents a densely related ingredient set. By visual observation, it is evident to see that ingredients of two types of functions (blue ones and purple ones, resp., act on targets in two disease-related target clusters) are mainly located in different modules. 

Since each edge of the network has its corresponding biology items in respect to GO processes, GO molecular functions, and GO localizations and pathways calculated by MetaDrug, that is to say, each interaction has its corresponding biology implications, ingredients of the same module must share certain GOs or pathways. We did analysis of these implications within each module to mine the underlying biology functions of the ingredient set. At last, the main functions of 3 largest modules were given out (as shown in Table 4). Take for example Module 2; most of the ingredients in this module have effects on the targets located in soluble fraction, cell projection, with the molecular functions of regulatory region nucleic acid binding, transcription factor binding, and protein binding, interfere with the processes of developmental process, regulation of cell death, and so on, and act on the main pathway of EGFR signaling pathways, IL-17 signaling pathways, Immune response_C5a signaling, and so on.

To make clear the main functions of the ingredients, we need to consider the three biggest modules. According to the colors of the nodes in these three modules, we can deduce that Module 1 is composed of some ingredients whose targets are in the disease-related target Cluster 5 and some ingredients whose targets are not in the disease-related target clusters, so it achieves therapeutic effects by influencing some processes which can contribute to the disease indirectly. Module 2 and Module 3 are composed of ingredients whose targets are mainly in the disease-related target Cluster 4 and Cluster 5, respectively; consequently they can restore the health state by acting on the disease-related targets.

To be detailed, we did further study and literature survey about the functions of the three main modules in Table 4.

The main function of Module 1 is related to steroid metabolism. In patients with steroid-resistant asthma, reduced steroid responsiveness is related to several molecular mechanisms. Nuclear translocation of glucocorticoid receptor (GR) *α* after binding corticosteroids is reduced, which might be due to phosphorylation of the GR activated by several kinases (p38 MAPK *α*, p38 MAPK *γ*, and c-Jun N-terminal kinase 1). Furthermore, increased expression of GR*β* competes with GR*α* and thus inhibits activated GR*α*, ultimately leading to steroid resistance. Long-acting *β*2-agonists can increase steroid responsiveness by reversing phosphorylation of GR*α*. Testosterone and 5*α*-dihydrotestosterone caused nuclear localization of 5-lipoxygenase, which initiated the biosynthesis of leukotriene and lipid mediators involved in asthma. Consequently, ingredients in Module 1 can contribute to the cure of asthma by the steroid metabolism. 

Ingredients in Module 2 were highly related to immune and inflammation processes and are core module for the therapy of asthma. Among the frequent pathways of Module 2, hyperactivation of EGFR signaling pathway was commonly involved in the mucous hypersecretion and initiated the activation of ERK1/2, PI3K, and Akt kinase in chronic inflammation of the airway [60]. In the pathway of angiotensin signaling via PYK2, PYK2 was essential for inflammatory cell migration and regulated airway inflammation, Th2 cytokine secretion, and airway hyperresponsiveness in the ovalbumin-sensitized mice during antigen challenge [61]. Vascular endothelial growth factor (VEGF) was secreted by human airway smooth muscle cells, and the level of VEGF was elevated by bradykinins through a protein kinase *C* and prostanoid-dependent mechanism [62]. The expression of VEGF and VEGFR in asthma patients increased accompanied by an increased number and size of blood vessels in asthmatic airways [63]. Interleukin-(IL-) 17 may inhibit the induction of tolerance to antigen through inducing IL-6 production, thereby suppressing the expansion of Foxp3-positive regulatory T (Treg) cells [64]. IL-5 is directly involved in recruiting eosinophils to the lung. Once recruited, eosinophils participate in the modulation of immune response, induction of airway hyperresponsiveness, and remodeling in asthma [65].

Function of module 3 is relevant to ion conduction and transport. The intracellular concentration of free ions regulates many cell functions such as secretion, contraction, motility, and transport processes. Ion channels including K and Ca channels modulated the activity of several structural and inflammatory cells and played an important role in the pathophysiology of asthma. These ion channels might serve as novel targets for the treatment of asthma [66]. Chloride channels are expressed on airway smooth muscle and influence airway smooth muscle force and cell length, which play an important role in the airway smooth muscle contraction and relaxation mechanisms. Activation of the ligand-gated chloride channel GABAA relaxed airway smooth muscle with the tachykinin and substance P. Activation of the glycine receptor was shown to relax airway smooth muscle with a selective neurokinin 2 receptor agonist [67]. 

Therefore, it can be inferred that different ingredients in the same module cooperate with each other to achieve certain effects; ingredients within each module act on targets with similar functions and play roles in related pathways. In the meantime, different modules with distinctive functions play different roles in the treatment to achieve overall effects by affecting different biological processes. 

In addition, we were also interested in the relationship between modules and herbs, and we got the related herbs for each module as shown in Table 5 by finding out herbs which are connected with the ingredients of the module in the herb-ingredient network (Figure 5). The result suggests that the ingredients of a module are mainly from different herbs. This result, together with the herb-ingredient network, can reveal that although different herbs have distinctive ingredients, they can collaborate with each other through ingredients with similar curative effects. 

### 3.6. The Mechanism of the Treatment of Feature Zhengs Based on the Module Functions

According to the network-based analyses of the ingredients of the key herbs, we infer that ingredients from different herbs can restore the healthy state by influencing multiple targets with different functions, and the ingredients share certain biological functions to collaborate with each other to obtain the therapeutic effects. By further mining the latent biology knowledge from the ingredient-network modules, the mechanism of the treatment of the feature Zhengs “FengXieFanFei,” “FeiQiShiXuan,” and “QiDaoLuanJi” was explained from three aspects.

1. The treatment has effects on the steroid metabolism which play a role in the treatment of asthma.
2. The treatment is highly related to immune and inflammation processes by influencing the pathways of EGFR, VEGF, Gn-RH, IL-17, and so forth, all of which play critical roles in asthma.
3. Regulation of ion conduction and transport, which can promote relaxation of airway smooth muscle and modulate the activity of structural and inflammatory cells, respectively, is another critical part in the treatment for the feature Zhengs.

## 4. Conclusions

TCM experts have accumulated many TCM clinical cases which contain meaningful treatment experiences and rules. To uncover the mechanism of treatment of Zhengs, systematical analyses about the clinical cases for patients with CVA have been done. The results can be shown from the following six aspects.

1. The feature Zhengs of CVA are “FengXieFanFei,” “FeiQiShiXuan,” and “QiDaoLuanJi.”
2. Eleven key herbs which not only play key roles by isolation analysis but also cooperate with each other frequently are SAA, Lum, PFL, PC, AGB, AT, FEJ, SC, PFS, RL, and FHE.
3. There are 18 functional clusters based on 372 reliable potential targets which are targeted by more than 7 compounds of the key herbs.
4. An herb-ingredient network was constructed, and we can catch sight of the composition features of herbs from the network.
5. An ingredient network, in which each ingredient was assigned functional items based on the enrichment analyses of potential targets in respect to GOs and pathways, was also constructed. It can present the functional relations of ingredients as well as getting the latent interaction mechanism.
6. Modules of the ingredient network which can show the latent collaboration among ingredients were identified, and main functions of the modules can help to illustrate the mechanism of the key herbs.

Herbal medicine is a complex system which can restore the state of patients back to a health level by influencing multiple bioprocesses [68]. The mechanism of the treatment of Zhengs which is highly related to the key herbs can be explained indirectly by analysis of the corresponding herbs.

In this work, we have discussed the functions of ingredients and targets of the key herbs and identified different target clusters and ingredient modules, which can provide evidence for explanations of the treatment of CVA. Since these key herbs are most commonly used for the treatment of the feature Zhengs “FengXieFanFei,” “FeiQiShiXuan,” and “QiDaoLuanJi,” the mechanism of the treatment of these Zhengs must agree with the actions of these key herbs. 

Based on the systematic analyses of key herbs, we have illustrated that the therapeutic effects of “FengXieFanFei,” “FeiQiShiXuan,” and “QiDaoLuanJi” were achieved by ingredients with similar functions in three main aspects as below.

1. The treatment has impacts on the metabolic processes of steroid.
2. The treatment is highly related to the immune and inflammation processes by influencing the pathways of EGFR, VEGF, Gn-RH, IL-17, and so on, all of which play critical roles in asthma.
3. Except for the two main functions above, the treatment also has effects on the regulation of ion conduction and transport.

In general, compared to Western drugs, TCM therapy aims to correct maladjustments and restore the self-regulatory ability of the body by influencing targets with multiple pathogenic effects. This systematical study provides a new conceptual framework for multilevel explanations and scientific understanding for the mechanism of TCM treatment and thus can promote the scientific understanding of TCM theory. However, considering the insufficiency of complete collections of the compositions of herbs and the prediction of targets, the mechanism still requires further rigorous studies. 

## Figures and Tables

**Table 1:** The number of compounds and potential targets.

Herb name	Number of compounds	Number of potential targets
SAA	19	494
Lum	23	447
PFL	15	303
PC	25	381
AGB	6	350
AT	6	8
FEJ	13	104
SC	29	29
PFS	4	428
RL	87	501
FHE	19	160

Number of nonrepetitive objects in total	223	954

**Table 2:** Gene functional clustering result.

Gene ID	Name	Gene ID	Name	Gene ID	Name
Cluster 1					
6257	RXRb	1958	EGR1	4520	MTF-1
10062	LXR-alpha	5468	PPAR-gamma	7376	LXR-beta
367	Androgen receptor	7342	LBP-1B	2626	GATA-4
2100	ESR2	10661	EKLF1	5914	RAR-alpha
8856	PXR	6667	SP1/SP3	6595	BRM
9971	FXR	6688	PU.1	2908	GCR-beta
2099	ESR1	6256	RXR-beta	4782	CTF
7067	TR-alpha	5458	POU4F2	2354	JunB
1386	ATF-2				

Cluster 2					
6196	p90RSK3	3706	IP3KA	29110	TBK1
1459	CSNK2A2	5599	MAPK8	5581	PKC-epsilon
8986	MSK2	1432	MAPK14	6195	p90RSK1
1017	CDK2	5594	MAPK1	5292	Pim-1
5587	PKC-mu	80271	ITPKC	5602	MAPK10
5580	PKC-delta	1021	CDK6	5347	PLK1
9252	MSK1	253430	IMPK	695	Btk
5601	MAPK9	6197	p90Rsk	9261	MAPKAPK2

Cluster 3					
28916	IGKV2-40	3500	IgG1	3502	IgG3
3501	IgG2	3494	IgA2	3514	IGKC
3497	IgE	3493	IgA1	3535	IgD
3503	IgG4				

Cluster 4					
718	C3	720	C4A protein	713	C1qb
3078	FHR-1	1191	clusterin	712	C1qa

Cluster 5					
9429	ABCG2	5243	MDR1	1080	CFTR
225	ALD2	5244	MDR3	23460	ABCA6
4363	ABCC1				

**Table 3:** Ingredients interactions.

Ingredient 1	Ingredient 2	GO processes	GO molecular functions	GO localizations	Pathways
Schaftoside	Glycyroside	Cellular aldehyde metabolic process, antibiotic metabolic process, and so forth	Alcohol dehydrogenase (NADP+) activity, Alditol:NADP+ 1-oxidoreductase activity, and so forth	None	Triacylglycerol metabolism p.1

Liquiritoside	Schaftoside	Aminoglycoside antibiotic metabolic process, daunorubicin metabolic process, and so forth	Alditol:NADP+ 1-oxidoreductase activity, oxidoreductase activity, and so forth		Triacylglycerol metabolism p.1

n-Hexanol	Glutamic-aci	Regulation of programmed cell death, regulation of multicellular organismal process, and so forth	Binding, regulatory region nucleic acid binding, and so forth	Neuron projection, axon, dendrite, and so forth	Immune response_CD40 signaling, Development_EGFR signaling pathway, Immune response_IL-3 activation and signaling pathway, and so forth

**Table 4:** Module functions.

Module	Aspect	Enriched items
1	GO process	Response to organic substance Lipid metabolic process Regulation of biological quality Hormone biosynthetic process Androgen metabolic process
	GO Molecular functions	Oxidoreductase activity Acting on the CH-CH group of donors Steroid dehydrogenase activity NAD or NADP as acceptor Catalytic activity
	GO localizations	Cell fraction Insoluble fraction Membrane fraction Cytoplasmic part Cell body fiber
	Pathways	Androstenedione and testosterone biosynthesis and metabolism Immune response_Gastrin in inflammatory response

2	GO process	Developmental process Regulation of cell death Response to organic substance Positive regulation of developmental process Response to stress
	GO molecular functions	Regulatory region nucleic acid binding Transcription factor binding Protein binding Regulatory region DNA binding
	GO localizations	Insoluble fraction Cell projection Dendrite Neuron projection
	Pathways	EGFR signaling pathways IL-17 signaling pathways Immune response_C5a signaling Immune response_IL-3 activation and signaling pathway Immune response_IL-5 signalling Immune response_IL-6 signaling pathway Apoptosis and survival_Role of CDK5 in neuronal death and survival Development_VEGF signaling via VEGFR2-generic cascades Mucin expression in CF via IL-6 Mucin expression in CF via TLRs

3	GO process	Aminoglycoside antibiotic metabolic process Synaptic transmission Gamma-aminobutyric acid signaling Inorganic anion transport Multicellular organismal process
	GO molecular functions	Alcohol dehydrogenase (NADP+) activity Ligand-gated channel activity Benzodiazepine receptor activity GABA receptor activity Chloride transmembrane transporter activity Passive transmembrane transporter activity
	GO localizations	Ion channel complex Chloride channel complex Neuronal cell body membrane Cell body membrane Synaptic membrane
	Pathways	Triacylglycerol metabolism p.1 Immune response_MIF-mediated glucocorticoid regulation

**Table 5:** Module-herb relation.

Module	Herbs
1	FHE, SAA, FEL, SC, and RL
2	PFL, PFS, SAA, Lum, PC, AGB, and RL
3	PFL, FHE, FEL, and RL
4	FHE
5	Lum, PC
6	FEL
